# Immunoglobulin E-Binding Pattern of Canadian Peanut Allergic Children and Cross-Reactivity with Almond, Hazelnut and Pistachio

**DOI:** 10.3390/biom10081091

**Published:** 2020-07-22

**Authors:** Mélanie Pitre, Lamia L’Hocine, Allaoua Achouri, Martin Blaquière, Anne Des Roches

**Affiliations:** 1 Saint-Hyacinthe Research and Development Centre, Agriculture and Agri-Food Canada, 3600 Casavant Blvd. W. Saint-Hyacinthe, QC J2S 8E3, Canada; melanie.pitre@canada.ca (M.P.); allaoua.achouri@canada.ca (A.A.); 2 Sherbrooke University Hospital Center, Hôtel-Dieu de Sherbrooke, Department of Pediatrics, 580 Bowen south, Sherbrooke, QC J1G 2E3, Canada; martin.blaquiere@usherbrooke.ca; 3 Immunoallergy and Rheumatology Service Department of Pediatrics Medical School CHU Sainte-Justine Institution, 3175 Côte Sainte-Catherine, Montreal, QC H3T 1C5, Canada; a.des.roches@umontreal.ca

**Keywords:** peanut, almond, hazelnut, pistachio, allergens, cross-reactivity, sensitization, inhibition ELISA, inhibition immunoblotting, IgE-binding, protein extracts

## Abstract

Peanut allergic individuals can be both co-sensitized and co-allergic to peanut and tree nuts. At the moment, standard diagnostic approaches do not always allow differentiation between clinically relevant sensitization and nonsignificant cross-reactions, and the responsibility of each allergen remains unclear. The objective of this study was therefore to determine a peanut sensitization profile in a cohort of Canadian peanut allergic children and assess the immunoglobulin E (IgE) molecular cross-reactivity between peanut, almond, hazelnut and pistachio. The specific IgE (sIgE) levels of each patient serum were determined by ImmunoCAP, indirect ELISA and immunoblot to examine their sIgE-binding levels and profiles to peanut proteins. Reciprocal inhibition ELISA and immunoblotting were used to study sIgE cross-reactions between peanut and the selected tree nuts using an adjusted and representative serum pool of the nine allergic patients. The results showed that the prepared peanut and tree nut protein extracts allowed for the detection of the majority of peanut and selected tree nut known allergens. The reciprocal inhibition ELISA experiments showed limited sIgE cross-reactivities between peanut and the studied tree nuts, with peanut being most likely the sensitizing allergen and tree nuts the cross-reactive ones. In the case of hazelnut and pistachio, a coexisting primary sensitization to hazelnut and pistachio was also demonstrated in the serum pool. Reciprocal inhibition immunoblotting further revealed that storage proteins (2S albumin, 7S vicilin and 11S legumin) could possibly account for the observed IgE-cross-reactions between peanut and the studied tree nuts in this cohort of allergic individuals. It also demonstrated the importance of conformational epitopes in the exhibited cross-reactions.

## 1. Introduction

Peanut and tree nuts are among the most potent food allergens. Allergic reactions to tree nuts and peanut are particularly associated with a higher degree of severity and are identified as leading causes of fatal induced anaphylaxis in North America [1,2]. Peanut and tree nut allergies are generally lifelong and seem to become more prevalent, particularly among children. In 2010, they were estimated to affect 1.68% and 1.59% of the Canadian population and 1.1% and 1.4%, of the United States population, respectively [3,4]. A more recent survey conducted between 2015 and 2016 estimates the prevalence of peanut allergies in children in the United States to be 2.5% [5], while Weinberger and Sicherer [2] reported an estimated tree nut prevalence varying between ~0.05% and 7.3%.

For those who are allergic to peanut or tree nuts, a stringent avoidance of the causal allergen and prompt treatment of reactions remains the cornerstone of treatment. In vitro cross-reactions between peanut and tree nuts are frequent. Maloney et al. [6] reported that 86% of 324 peanut-allergic patients were sensitized to tree nuts, but only 34% were clinically allergic to tree nuts. Cross-reactivity often occurs between allergenic molecules in closely related species or well-preserved molecules with similar functions present in widely different species, such as storage proteins, including vicilins, 2S albumins and globulins. However, it has also been described between phylogenetically distant species. In these cases, the allergens responsible are usually homologous proteins belonging to specific families of molecules. Frequently, the reaction is caused by proteins that are highly conserved from an evolutionary point of view and that, given their widespread presence, have been termed panallergens, such as the prolamin, PR-10, profilins and lipid transfer protein families [7]. 

Homologous proteins share different degrees of sequence identity, similar 3D-structures and common epitopes recognized by immunoglobulin E (IgE) antibodies [8]. The presence of IgE cross-reactivity between peanut allergens and tree nuts has been reviewed by several recent studies [2,9,10,11], but the identification of the individual cross-reactive allergens is rather limited. In an earlier study, de Leon et al. [12] demonstrated serum IgE cross-reactivity between allergens present in peanut, almond, Brazil nut and hazelnut. By using bioinformatics approaches, some structural homology was observed between surface-exposed epitopes on legumins and vicilins, which could contribute to the cross-reactivity seen between peanut and tree nut allergens [13,14]. Interestingly, when using BLAST protein comparison tool to compare peanut and tree nut allergen amino acid sequences obtained from PubMed protein searches, none of the peanut allergens have a sequence identity greater than 70% with any tree nut [11]. It is generally estimated that at least 70% overlap is required for cross-reactivity to be significant [15]. However, de Leon group has shown by using inhibition ELISA and basophil activation tests that peanut-specific IgE can cross-react with almond, Brazil nut and hazelnut allergens, and these cross-reactive IgE antibodies were biologically active [16]. In a later study, it was demonstrated that Brazil nut and almond extracts inhibited the binding of IgE in a peanut-allergic serum to Ara h 2, indicating the presence of shared IgE-binding epitopes between Ara h 2 and proteins from these tree nuts [17]. In many cases, the responsibility of each allergen in the cross-reactivity has not been clearly defined. 

Peanut-allergic individuals can be both co-sensitized and co-allergic to peanut and tree nuts. At the moment, the practice of avoiding all tree nuts for peanut-allergic patients is changing as a result of the recognition that both skin prick testing (SPT) and serum-specific IgE (sIgE) testing of tree nut allergies lead to many false-positives, resulting in the unnecessary avoidance of tree nuts and does not always allow differentiation between clinically relevant sensitization and nonsignificant cross-reactions [18]. The discussion is continuously ongoing on the immunological phenomenon of cross-reactivity and its role for the diagnosis, management and treatment of certain food allergies. Further studies are therefore still needed to help answer the question whether peanut-allergic individuals should systematically avoid tree nuts. This is particularly pertinent, as several recent studies based on oral food challenges found less than 30% of coexistent tree nut allergies in peanut-allergic patients compared with studies based on SPTs and/or sIgE results [19]. In general, the decision to perform oral challenges is based on clinical history and the results of the SPTs and/or sIgE results [10]. In the case of positive SPT or IgE tests, a specific tree nut should be introduced via a physician-supervised food challenge, whereas, if both SPT and sIgE are negative and there is no history of ingestion/reaction, a home introduction is recommended [20]. Thus, although in vitro sIgE testing cannot be a diagnostic test alone for food allergies [21], it remains a valuable tool for assessing sensitization and evaluating the likelihood risks of cross-reactions prior to skin-prick testing and clinical oral challenges. Moreover, data on sensitization profiles to peanut and tree nut allergens in Canadian peanut-allergic children, as well as on molecular cross-reactivity patterns to commonly locally consumed tree nuts, are limited. Hence, this study was conducted with the objective to bring insight into peanut sensitization profiles in a cohort of Canadian children with confirmed clinical allergies to peanut and assess the sIgE molecular cross-reactivity between peanut, almond, hazelnut, and pistachio using reciprocal inhibition ELISA and immunoblotting. 

## 2. Materials and Methods

### 2.1. Materials

Raw peanut (*Arachis hypogea*), almond (*Prunus dulcis*), hazelnut (*Corylus avellana*) and pistachio (*Pistachia vera*) were purchased from local wholesale markets in Montreal, QC, Canada. The seeds were hand-cleaned to remove any broken seeds or foreign material. The seeds were then shelled, skinned and stored under vacuum at 4 °C until use.

### 2.2. Human Sera from Allergic and Nonatopic Subjects

Human sera samples were provided by 9 young patients with a clinically confirmed allergy to peanut. The sera were collected at the Sainte-Justine University Hospital Center (Montreal, QC, Canada). Levels of sIgEs for peanut and tree nuts studied were measured by means of the Pharmacia ImmunoCAP system (Phadia, Uppsala, Sweden). Skin-prick tests (SPTs) to raw peanut extracts were performed to all patients using a sterile 25-gauge needle according to the recommended protocol [22]. Positive and negative controls were performed using histamine 10 mg/mL and saline 0.9%, respectively. The skin test site was measured after 15 min, and a positive result was recorded if the mean wheal diameter was at least 3 mm larger than that of the negative control and at least 50% greater than that produced by the positive control.

Three healthy nonallergic subjects with low total IgE response were selected as a nonatopic control group. Sera from three subjects with allergies to dust mites were used as a negative control group and purchased from Plasma Lab International (Everett, WA, USA). All sera were frozen at −80 °C until use. This study was approved by the Sainte-Justine’s Hospital Ethics Committee (3225/2013). An informed written consent was obtained from parents of all participants. All manipulations involving the use of human sera were carried out at the specialized laboratory of Saint-Hyacinthe Research and Development Centre (Saint-Hyacinthe, QC, Canada) using universal precaution (UP) normalized practices. 

### 2.3. Preparation of Defatted Peanut and Tree Nuts Flours

Peanut and tree nut seeds were frozen in liquid nitrogen and ground in an analytical mill (IKA A11, IKA, Staufen, Germany) in order to obtain a fine and homogeneous powder. Full fat flours (30 g) were extracted with 5 volumes of hexane, with constant magnetic stirring. The slurry was filtered through a filter paper under vacuum, and extractions were repeated three times. Defatted samples were dried overnight (~10‒12 h) in a fume hood in order to remove all traces of residual solvent. Defatted flours were then homogenized for 30 seconds in a coffee grinder (Custom Grind Deluxe, Hamilton Beach, Washington, NC, USA) and stored in screw-capped plastic tubes at −80 °C until further use.

### 2.4. Preparation of Crude Protein Extracts from Peanut and Tree Nuts

Each nut was extracted under optimized buffer conditions, as previously reported by L’Hocine and Pitre [23]. Briefly, defatted peanut and almond flours were extracted in carbonate buffer 0.05 M, pH 9.6 at ionic strengths of 0.075 and 0.15, respectively. Hazelnut were extracted in phosphate buffer 0.03 M, pH 7.2 at an ionic strength of 0.5, while pistachio were extracted with borate buffer 0.2 M, pH 8.5 at an ionic strength of 0.15. Each extraction was done at a protein sample-to-buffer ratio of 1:250 (w/v) by weighing 0.4 g of defatted peanut and pistachio flours or 0.5 g of almond and hazelnut flours in a 50-mL centrifuge tube and adding, respectively, 47, 40, 48 and 39 mL of the appropriate buffer. Extractions were done at room temperature for 1 h. All extractions were conducted under constant shaking at 45 rpm by using an agitator (Lab Roller, Labnet, Edison, NJ, USA) inclined at a 45-degree angle. The crude extracts were then transferred into 70-mL centrifuge bottles and centrifuged (Beckman J2-21, Life Sciences Division, Indianapolis, IN, USA) at 16,000 g for 30 min at 4 °C. The supernatant was vacuum-filtered (Whatman filter paper No. 4, Whatman International Ltd, Maidstone, England). Clarified extracts were transferred in 2-mL cryogenic vials and stored at −80 °C. 

### 2.5. Protein Quantitation

Protein content in the defatted flour samples was determined by Dumas combustion (Leco FP-428, Leco Corporation, St. Joseph, MI, USA). The protein percentage was calculated from the percent nitrogen content using the conversion factors proposed by Venkatachalam and Sathe [24], as follows: 5.18 for almond, 5.46 for peanut, and 5.3 for hazelnut and pistachio. Soluble proteins in peanut and nut extracts were determined by the Bradford method (Bio-Rad protein assay, Bio-Rad Laboratories, Hercules, CA, USA) using bovine serum albumin (BSA) as the standard protein in the appropriate buffers.

### 2.6. Protein Electrophoresis 

Peanut and tree nut soluble extracts were separated by SDS-PAGE. The extracts were mixed with an equal volume of Laemmli sample buffer (62.5-mM Tris-HCl (pH 9.6), 25% glycerol, 2% SDS, 5% ß-ME and 0.01% bromophenol blue) and boiled for 5 min. Nine micrograms of protein/well for peanut, almond, hazelnut and pistachio were loaded onto TGX AnyKD gels (Mini Protean, Bio-Rad) and electrophoresed on a Criterion cell (Bio-Rad). The gels were stained with Coomassie Brilliant Blue. Stained gels were scanned and calibrated by using an Image Scanner III (GE Healthcare, Amersham, UK) operated by LabScan 6.0 software (GE Healthcare). For the image analysis, the Image Quant TL 7.0 Software (GE Healthcare) was used.

### 2.7. Serum Immunoglobulin E (IgE) Indirect ELISA 

For IgE indirect ELISA, raw peanut protein extract (0.25 µg/well) was coated on 96-well polystyrene plates with a high binding surface (Costar™, Corning, NY, USA) in coating buffer (0.05-M carbonate bicarbonate buffer, pH 9.6) and incubated overnight at 4 °C. Negative controls, nonatopic controls and a blank sample for nonspecific secondary antibody binding were included. Absorbance values ˃ 0.1 optical density (OD) units (mean plus 3 x SD of negative control sera) were considered positive. All subsequent incubations were performed at 25 °C on a microplate shaker (Jitterbug, Boekel Scientific, Philadelphia, PA, USA). The following day, the coated wells were blocked using 150 µL/well of 5% bovine serum albumin (BSA) in 10-mM sodium phosphate (pH 7.4) with 137-mM sodium chloride (NaCl) and 0.1% Tween-20 (PBS-T) for 2 h. The microplates were washed in PBS-T and then incubated with 50 µL of 1:128 dilutions of sera and incubated for 2 h. The wells were washed and then incubated with 50 µL/well of 1:3000 dilution of peroxidase-labeled mouse anti-human IgE- fragment crystallizable-horseradish peroxidase (Mouse Anti-Human IgE Fc-HRP) (Southern Biotech, Birmingham, USA) in a dilution buffer (1% BSA in PBS-T). After incubation for 1 h, the bound peroxidase activity was determined with tetramethylbenzidine (TMB) (50 µL/well, Sigma-Aldrich, Oakville, ON, Canada), the reaction was stopped with 1 N H_2_SO_4_ (50µL/well) and absorbance was measured at 450 nm. The measurements were performed in triplicate.

### 2.8. Serum IgE Indirect Western Immunoblotting 

For the IgE indirect immunoblotting, peanut and tree nut protein extracts were first separated by SDS-PAGE as described above and then transferred onto a polyvinylidene difluoride (PVDF) membrane (Immun-Blot, Bio-Rad, Hercules, California, USA) using a Mini Trans Blot (Bio-Rad) and Towbin transfer buffer. The transfer was performed at 100V for 60 min—after which, the membrane was recovered and air-dried. The membrane was then blocked in 3% (w/v) skim milk powder for 1h at room temperature and divided into strips. The peanut and tree nut protein immobilized strips were incubated overnight at 4 °C with a 1:50 dilution of the individual serum or a pool of sera from the nine peanut-allergic patients. After washing, the membrane strips were incubated for 1h at room temperature with a 1:10000 dilution of peroxidase-labeled mouse anti-human IgE (Southern Biotech 9160-05). They were then washed extensively and developed with amplified Opti-4CN reagents (Bio-Rad) following the recommendations of the manufacturer. The immunoblots were scanned and analyzed as described above for the SDS-PAGE gels. 

### 2.9. Reciprocal IgE Inhibition ELISA and Immunoblotting

Reciprocal inhibition ELISA and immunoblotting tests for peanut and tree nuts were performed using pooled sera from the nine pediatric patients. Individual sera were pooled according to their level of sIgE-binding responses in indirect ELISA; the higher the response, the lower the volume used in the pool. Inhibition ELISA experiments were performed by coating 96-well microplates with peanut or tree nut protein extracts in coating buffer (0.05-M carbonate bicarbonate buffer, pH 9.6) and incubated overnight at 4 ℃. Diluted serum pool was preincubated overnight at 4 °C with each inhibiting extract in a concentration range of 0.00001 to 100 µg protein/mL. On the following day, the coated plates were washed with PBS-T and blocked with a solution of 5% BSA in PBS-T (10-mM sodium phosphate (pH 7.4) with 137-mM NaCl and 0.1% Tween-20), as described above. After washing, the inhibitor mixtures were transferred to the peanut or tree nut-coated plates (50µL/well) and incubated for 2 h at 25 °C. Secondary antibody incubation and detection were performed as described above for the indirect ELISA test. Pooled sera with no inhibitors were used as the positive control (100% reactivity). Percentage inhibition was calculated according to: (1 – (OD450 serum with inhibitor/OD450 serum without inhibitor)) x 100. Concentrations of the different peanut or nut extracts that inhibited 50% of the IgE binding to the coated protein (IC_50_) were determined whenever possible. Reactions with more than 15% inhibition over the background were considered positive. All the determinations were carried out as duplicates. 

For reciprocal inhibition immunoblotting, experiments were conducted as described above for the indirect immunoblotting assay, except that peanut immobilized membrane strips were incubated with the pooled sera (diluted 1:25) that was first preincubated with an equal volume of either almond, hazelnut or pistachio extracts (200 µg of protein) overnight at 4 °C. For almond, hazelnut or pistachio-immobilized trips, they were incubated with a pooled sera (diluted 1:8) that was first preabsorbed with the peanut extract (200 µg of protein). In the experiments, immunoblots of ovalbumin-inhibited sera pools were used as a negative control, and uninhibited sera were used as a positive control. Inhibition with serum from a non-peanut and non-tree nut atopic control was also included. 

### 2.10. Statistical Analysis

Each experiment was run in triplicate, and the data were expressed as means ± standard deviation. Statistical analyses were performed using XLSTAT software (Addinsoft, New York City, NY, USA) in Microsoft Excel (Redmond, WA, USA). One-way analysis of variance (ANOVA) and the Tukey’s honest significant difference (HSD) test (*p* < 0.05) were performed to detect significant differences.

## 3. Results and Discussion

### 3.1. Clinical Profiles of Peanut-Allergic Patients and their Sensitization Profiles to Tree Nuts by IgE ImmunoCAP Testing

Table 1 summarizes the clinical reactivity profiles of the peanut-allergic children’s results of their skin-prick tests for peanut, as well as their sensitization profiles to tree nuts. The ImmunoCAP system (CAP) was used in the evaluation of specific IgE (sIgEs) levels for peanut, almond, hazelnut and pistachio in patients’ sera. Allergen-specific IgE titers above 0.35 kUA/L were considered positive.

The reported severity of the clinical allergic reactions to peanut in the studied cohort of allergic children varied from mild urticaria to potentially lethal anaphylactic ones. Peanut skin-prick testing ranged from 6 to 15 mm, and peanut-specific IgE (sIgE) levels were superior to 100 kU/L for all patients. It is generally considered that peanut SPT ≥ 8 mm and peanut sIgE ≥ 15 kU/L have 95% positive predictive values for challenge-proven peanut allergies [25,26]. The concentration of sIgE to almond, hazelnut and pistachio varied among the allergic children, revealing different sensitization levels to the studied tree nuts (Table 1). Specific IgE levels to almond and pistachio were < 15 kU/L for all patients. Three patients had superior sIgE levels towards hazelnut of 16, 60 and > 100 kU/L. One patient showed no sensitization to tree nuts by ImmunoCAP testing. 

Peanut (*Arachis hypogaea*) belong to the legume family (*Leguminosae*). They are botanically related to soybean, bean and pea but not to tree nuts. However, it has been commonly reported that patients with peanut allergies showed a strong sensitivity to distantly related tree nuts allergens [6,27,28]. The molecular basis for the observed coexisting sensitivities is not yet fully understood, as it remains unclear if they are the result of multiple primary sensitivities or molecular cross-reactivities. Major allergens in peanut and tree nuts belong to a relatively small number of protein families, particularly storage proteins (2S albumins, vicilins, legumins and profilins) [7], indicating that conserved structures and biological activities play a role in determining or promoting the sensitizing and cross-reactivity properties of proteins [29,30].

### 3.2. Electrophoretic Protein Profiles of Prepared Peanut and Selected Tree Nut Extracts and Correspondence to Known Allergens 

**Figure 1:**
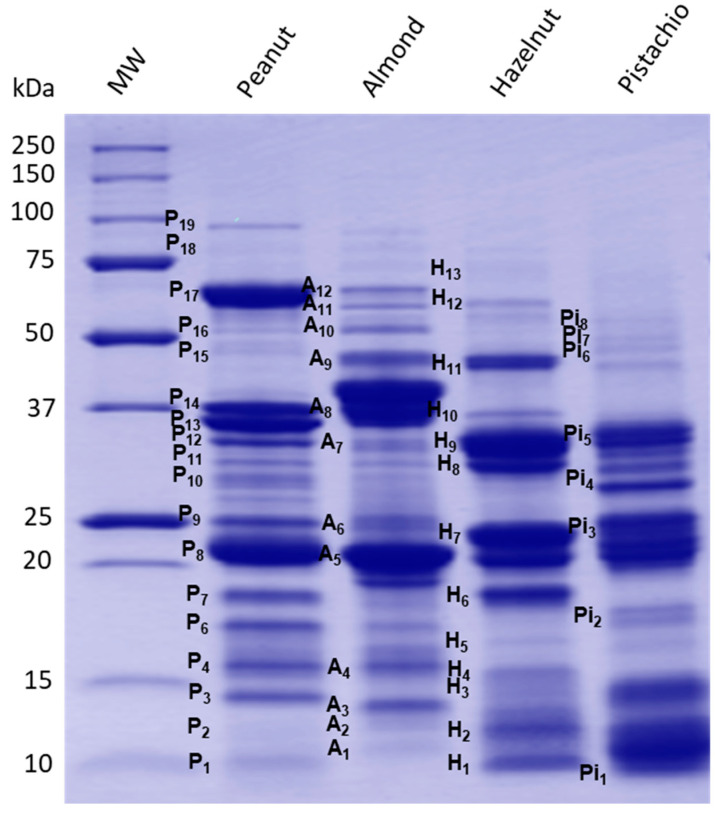
Reduced SDS-PAGE profile of peanut, almond, hazelnut and pistachio protein extracts.

Protein profiling of the prepared peanut and tree nuts extracts were carried out by SDS-PAGE analysis (Figure 1), and major allergen polypeptides were described based on information available on peanut and tree nut known allergens in the database of the International Union of Immunological Societies [31] and the published literature (Table 2). As shown in Figure 1, clear differences were observed in the resolved protein banding patterns between peanut and other tree nut extracts. Several protein bands ranging from 10 to 100 kDa were detected. Many prominent protein bands of each peanut and tree nuts have been described as potent allergens and classified in current allergen databases and published literature.

Peanut is one of the best-studied food allergens. To date, 17 allergenic proteins have been identified and registered by the World Health Organization/International Union of Immunological Societies (WHO/IUIS) subcommittee on the nomenclature of allergens [31] (Table 2). In the case of almond, hazelnut and pistachio, the number of characterized allergens is relatively more limited (Table 2). Many of the identified peanut allergens have protective functions or are seed storage proteins [32]. The densitometric analysis of the electrophoretic profile of the peanut protein extract showed the presence of several peanut bands named herein P3, P4, P6, P7, P8 and P9, which were described as potent allergens. Thus, P3 (14.08 kDa) and P4 (15.7 kDa) bands have been described as Ara h 6 and Ara h 7, which are storage proteins of the 2S albumin type (conglutin). The amino acid sequence of Ara h 6 is 53% similar to Ara h 7, and both share 59% and 35% sequence identity and secondary and tertiary structure homology, as well as immune cross-reactivity with Ara h 2 [33]. Yet, little is known about Ara h 6 and Ara h 7. Bands P6 (17.20 kDa) and P7 (18.50 kDa) exhibited similar molecular features to Ara h 2, consisting of two isoforms—namely, Ara h 2.01 and Ara h 2.02—with reported molecular weights of 17 and 19 kDa. Ara h 2.02 is reported to be a more potent allergen compared to Ara h 2.01 [34]. Bands P8 (21.48 kDa) and P9 (24.95 kDa) could possibly belong to fragments of the 11S storage protein from the glycinin family. These bands correspond to the allergenic protein Ara h 3/4, consisting of a basic subunit [35]. The two subunits are covalently linked by an intermolecular disulfide bridge and associate into a very stable hexameric structure of the 11S glycinin [36]. P10 to P14 bands (molecular weight varying from 29-37 kDa) are also described as acidic fragments of 11S, which derive from the proteolytic process of the oligomeric structure of the 11S globulin protein (Ara h 3). Additionally, P17 corresponds to the allergen Ara h 1, which is a 62.33-kDa glycoprotein assembled in di- and trimeric complexes that has been well-described [37,38]. The band P19 (96 kDa) could possibly correspond to the lipoxygenase enzyme, which has been reported in peanut samples [39]. However, no information was reported in the literature about peanut lipoxygenase as a potent allergenic protein. Finally, less-abundant bands around 10 kDa (P1), 12.04 kDa (P2), 16.22 kDa (P5) and 51.73 kDa (P16) were also present in the peanut extract. Some of these polypeptides may correspond to other peanut allergens, such as Ara h 9 (nonspecific lipid-transfer protein type, nLTP), Ara h 5/Ara h 11 (profilin/oleosin), Ara h 10 (oleosin) and Ara h 3 (11S globulin), respectively, which were reported with similar corresponding molecular weight ranges [32]. 

For the almond extract, the protein SDS–PAGE profile (Figure 1) revealed the presence of several bands that were described as major almond allergens. Presently, eight allergenic proteins have been identified and characterized in almond according to their biochemical functions. However, only six, Pru du 3, Pru du 4, Pru du 5, Pru du 6 (amandin), Pru du 8 and Pru du 10, are recognized and included in the WHO/IUIS list of allergens [31]. Most of these reported allergens may correspond to protein bands present in the electrophoretic pattern. The SDS-PAGE analysis of the almond extract showed a particularly high abundance of A5, which has been described as amandin, a potent almond allergen named Pru du 6. Amandin is the major storage protein in almond, which is composed of two polypeptides with estimated molecular weights of 42–46 kDa (and 20–22 kDa linked via disulfide bonds) [40]. The gel showed, also, several protein bands that are in agreement with the molecular features of known allergens [41]—namely, A1 was described as Pru du 5 (ribosomal protein), A2 as Pru du 2S albumin, A3 as Pru du 4 (profilin), A4 as Pru du 1 (pathogenesis-related protein, PR10), A6 as Pru du 2 (thaumatin-like protein, PR5), A7 as Pru du 8 (antimicrobial seed storage protein), A9 as Pru du γ-conglutin and A11 as Pru du 10 (mandelonitrile lyase 2). These allergens were previously isolated, identified and fully characterized [42,43,44,45,46].

The protein extract of hazelnut revealed the presence of eight major polypeptides with a MW of 10.2, 11.72, 15.15, 18.46, 23.25, 33.03, 45.04 and 60.28 kDa. Based on their molecular features, these polypeptides correspond to currently known allergens from hazelnut—namely, H1 to Cor a 8 (nsLTP,), H2 to Cor a 14 (conglutin 2S albumin), H4 to Cor a 13 (oleosin), H6 to Cor a 1 (Bet v 1-homolog), H7-H10 to Cor a 9 (legumin) and H11/H12 to Cor a 11 (vicilin 7S globulin). Faint bands were also observed on the gel, with estimated MW of 14.2 kD, 16.14 kDa and 74.25 kDa, which might correspond to other hazelnut allergens, such as Cor a 2 (profilin, H3), Cor a 12 (oleosin, H5) and Cor a 10 (luminal-binding protein, H13), respectively. All these allergens have been well-documented in the literature [47,48] and in allergen online databases [32]. For pistachio, the SDS–PAGE revealed several bands from 10 to 15 kDa and 20 to 37 kDa, as well as a less-abundant doublet at 17 kDa and between 45 to 60 kDa. The molecular features of these bands are compatible to a number of pistachio allergens that have been isolated and identified. The Pi5 (36 kDa) might be associated with Pis v 5 [31], which is suspected to have an important role in the development of pistachio nut hypersensitivity [49]. Other resolved pistachio protein bands Pi1/Pi2 may correspond to Pis v 1 (2S albumin, 7 and 17 kDa); Pi3 band for Pis v 4; Pi4, Pi6 and Pi7 to Pis v 2 subunits, a superoxide dismutase of 26 kDa and Pi8 band for Pis v3 [50,51,52]. In addition, the location and molecular weight of the SDS-PAGE-resolved pistachio bands agreed well with the pistachio allergen identifications by liquid chromatography-mass spectrometry (LC/MS/MS) recently reported by Vicente et al. [53]. Moreover, the SDS-PAGE analysis of the prepared extracts showed that the utilization of optimal nondenaturing protein extraction conditions that are specific to peanut and to each selected tree nut [23] allows for a representative extraction of allergens, thereby facilitating a comprehensive investigation of sIgE-binding to a majority of peanut, almond, hazelnut and pistachio allergens. This is further supported by the findings of Sanchiz et al. [54], who obtained similar SDS-PAGE profiles by both the direct solubilization of raw pistachio flours in denaturing SDS Laemmli sample buffer to obtain the total proteins and by direct extraction in borate-buffered saline to obtain the soluble protein extracts. The protein-denaturing conditions of the Laemmli sample buffer, however, may not allow an accurate representation of the allergen IgE-binding response naturally present in the source material—particularly for the ELISA assay, where a preserved native structure of the protein is desired for the optimal detection of conformational epitopes.

### 3.3. Indirect ELISA and Immunolotting for IgE Responses to Peanut Proteins

**Figure 2:**
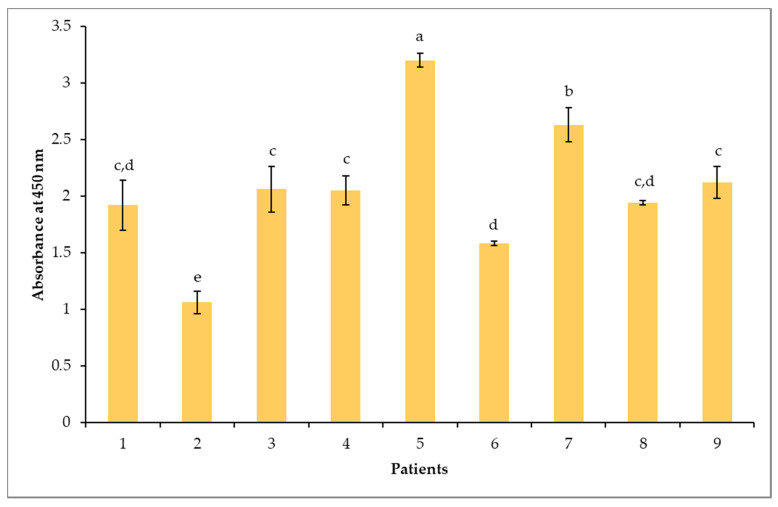
Indirect ELISA analysis of specific immunoglobulin E (IgE)-binding to the raw peanut extract for each individual patient. Different letters represent statistical differences according to Tukey’s test, with a confidence level of 95%. Values represent means ± SD (*n* = 3).

An indirect ELISA test was performed with each individual serum to confirm and evaluate the sIgE-binding levels to the immobilized raw peanut protein extract. As shown in Figure 2, all sera were positive towards the raw peanut extract. Interestingly, unlike ImmunoCAP testing, the ELISA test revealed significant (*p* < 0.05) differences in the intensity of the responses among the tested sera. The binding response of sera from patients 2 and 6 to the peanut protein extract was less pronounced, while sera from patients 5 and 7 revealed the strongest sIgE binding. 

**Figure 3:**
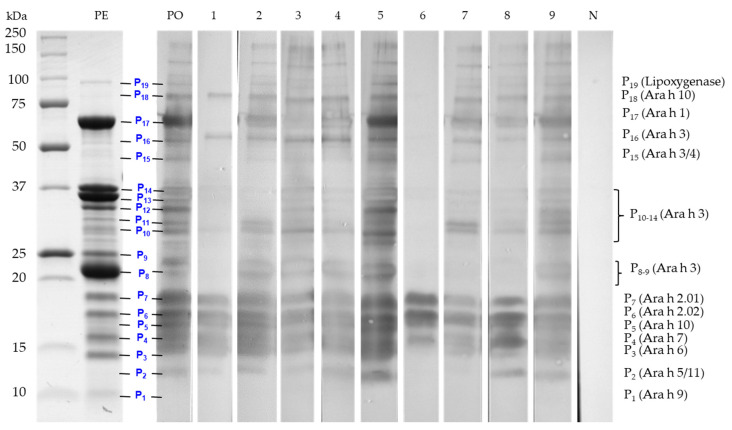
Electrophoretic profile of peanut (PE) and IgE immunoblots of peanut protein extracts with a serum pool (PO) of 9 peanut allergic patients and with each individual patient’s serum (1 – 9). A mite negative serum (N) was used as a control.

The differences in the sIgE ELISA-binding response of the sera was used to establish a representative pool of sera. The volume contributed by each patient’s serum to the pool was adjusted according to the observed intensity of sIgE binding. The contributing volume of the less-intense serum was higher in the pool than the one with a higher response. This adjustment avoids that the pool behaves like a dilution of the strongest serum, thereby hiding protein binding to the low sIgE titers from the other sera [8]. The pattern of IgE-binding of the pool (PO) and the nine individual sera to the peanut protein extract was determined by immunoblotting. As shown in Figure 3, clear differences were observed in the resolved protein IgE-binding profiles among the tested sera. The majority of children presented intense bands primarily reacting in the regions of 12-19 kDa, 20-25 kDa, 30 to 40 kDa and 45 to 250 kDa, corresponding to a majority of the resolved proteins (P1-P19) in Figure 1 and Table 2. Of these, the protein bands compatible to Ara h 1, Ara h 2, Ara h 3, Ara h 6, Ara h 7 and Ara h 10 appear to be the most sensitizing peanut allergens for all nine allergic children (Figure 3). A strong reactivity to Ara h 2 and Ara h 6 was observed for all nine allergic patients, confirming once more the importance of sIgE against Ara h 2 and Ara h 6 as the highest predictors of clinical reactivity to peanut [79]. Patients 1, 6, 7 and 8 showed, however, no or very little reactivity toward the Ara h 3/4 glycinin subunits around 22–24 kDa. In addition, an IgE-binding band named P2, which was not apparent on the SDS-PAGE Coomassie blue coloration (Figure 1), was detected in the immunoblots of all patients, except patients 6 and 7, who lacked reactivity to this band (Figure 3). This protein band (P2) could possibly be attributed to Ara h 5 (profilin, 12-15 kDa [56]) and to Ara h 11 (oleosin, 14 kDa [32]) (Table 2). Moreover, patient 6 had a different sensitization pattern, since he only showed IgE-binding in the 15-20 kDa region corresponding herein to P4-P7, which was associated to Ara h 7, Ara h 10, and Ara h 2 (Table 2). Finally, sera of seven allergic patients (except patients 1 and 6) showed IgE binding to protein bands above 100 kDa (Figure 3). These bands could correspond to reported dimeric and trimeric isoforms of Ara h 1 [80]. Sera from low IgE and from dust mite-allergic patients that were used as negative controls (last strip) did not react with any peanut protein bands. Furthermore, Figure 3 showed that the adjustment of the volumes contributed by each patient’s sera to the observed IgE-binding intensities allowed for the preparation of a pool of sera (PO) that reacted representatively of all nine sera, showing the most prevalent and less prevalent IgE-reactive bands. For example, band P11 (corresponding to the Ara h 3 fragment), which is reacting to the sera of patients 2 and 7 but not to the more intense serum of patient 5, appeared in the pool immunoblot. Utilization of the pooled sera has both advantages and limitations. One of the advantages is that it will rule out the identification of inter-individual differences; however, it might also average the IgE-binding response and, in this way, reduce the detectability. By readjusting the volumes contributed by each patient’s serum, where the volume of less intense sera is higher in the pool than one with a higher response, will generally improve the detectability [8]. 

Peanut allergies differ clinically and immunologically in different areas of the world, probably due to differences in exposure [81]. Thus, Cabanillas et al. [81] reported in their review that peanut-allergic patients from New York included in a study were sensitized to Ara h 1, Ara h 2 and Ara h 3 in high proportions (56.7% to 90%) and had severe symptoms to peanut [82], whereas Ara h 8 and Ara h 9 have been demonstrated to be major allergens in Central/Western and Southern Europe, respectively [82,83]. Spanish patients (both pediatric and adults) typically developed peanut allergies after becoming allergic to other foods derived from plants such as peaches [83]. The highest sensitization rate to the Bet v 1 homolog Ara h 8, as well as to Bet v 1, was also found in Swedish patients, which reflects the high birch pollen exposure in that country [83]. 

The pattern of sensitization to peanut allergens was also reported to be dependent on age [81]. Several European studies showed that sensitizations to Ara h 1, Ara h 2 and Ara h 3 were frequently acquired in childhood and rarely in adolescence and adulthood, while sensitizations to Ara h 8 and Ara h 9 were linked to late-onset peanut allergies, with the expectation that an increased exposure to pollen occurs as an individual matures [81,82,84,85]. This finding has been further confirmed by a recent study by Valcour et al. [86], who reported a more prevalent sensitization to peanut allergens Ara h 2, Ara h 1 and/or Ara h 3 across the Unites States in infants, while positivity to Ara h 8 was relatively low for young children and increased with age for adolescents and young adults [86]. Our results are in-line with these observations, as the studied cohort of Canadian peanut-allergic children exhibited a typical North American sensitization profile, with intense reactivity to major peanut storage proteins Ara h 2, Ara h 1 and/or Ara h3 [82,86], the most prevalent sensitizations being towards Ara h 2 and Ara h 6, which have been associated with severe/systemic reactivity in peanut-allergic individuals [10,87]. 

### 3.4. Evaluation of IgE Cross-Reactivity Between Peanut and Tree Nuts by Reciprocal Inhibition ELISA

**Figure 4:**
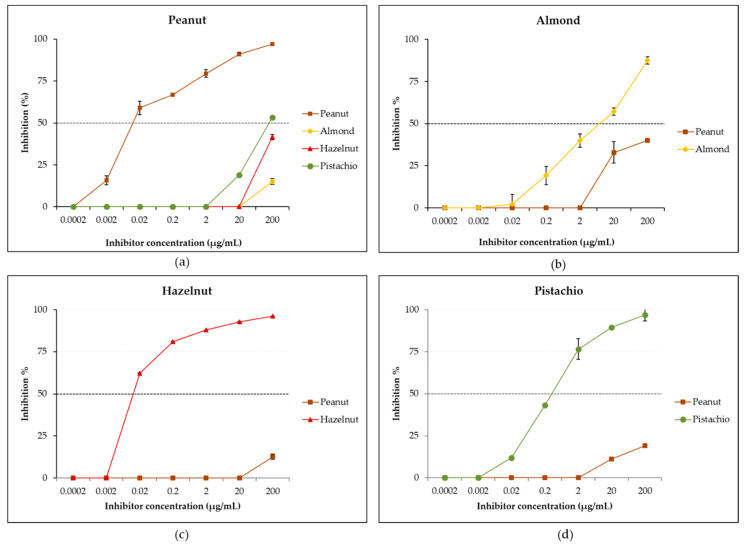
Reciprocal IgE inhibition ELISAs with peanut (**a**), almond (**b**), hazelnut (**c**) and pistachio (**d**) extracts adsorbed to the solid phase with inhibitor protein concentrations from 0.0002 to 200 µg/mL. The dashed horizontal line intercepts the inhibition curves at the median inhibitory concentration (IC_50_).

IgE cross-reactivity between peanut, almond, hazelnut and pistachio was first studied by reciprocal inhibition ELISA using a pool of sera due to the limited available volume for each individual serum. As shown in Figure 4a, when the peanut was immobilized in the solid phase and the pooled sera preincubated with tree nut extracts, only a partial inhibition to peanut was observed. Thus, preincubation with the almond extract led to a negligible inhibition of 15% of IgE-binding to peanut at the maximal inhibitor concentration of 200 µg/mL, while the hazelnut extract resulted in 42% inhibition and the pistachio extract in a higher partial inhibition of 53%. A complete inhibition (100%) of IgE-binding was obtained when the pooled sera was preincubated with the peanut extract. These results indicate the presence in the sera of a majority of sIgEs that recognize peanut proteins and which do not bind to the studied tree nut proteins, suggesting a moderate level of cross-reactivity between peanut and tree nuts. The mild percent inhibition obtained with tree nut extracts confirms the presence of a weak cross-reactivity with pistachio proteins, followed by hazelnut and then, in a negligible way, with almond proteins. However, when studying reverse inhibition—that is, when the various tree nuts were the solid-phase allergens and the peanut extract, the inhibitor—a partial inhibition of 40% was recorded for almond (Figure 4b), suggesting the presence of sIgEs that uniquely bind to almond but, also, of sIgEs that cross-reacted with peanut proteins. As for hazelnut (Figure 4c) and pistachio (Figure 4d), only negligible inhibitions of 13% and 19% were obtained, respectively, suggesting the presence in the pool of sIgEs that uniquely bind to pistachio and hazelnut and that do not cross-react with peanut allergens. The study of reciprocal inhibition allows to analyze the relationship between two cross-reactive allergens and to evaluate the extent of the affinity of IgE-binding to the allergen, as well as to ascertain if copositive IgE reactions are the result of immunological cross-reactivity or co-sensitizations [8]. Co-sensitization describes the presence of sIgE towards distinct and unique epitopes in different allergen sources, while cross-reactivity refers to the presence of sIgEs that recognize homolog molecules from different allergen sources [88]. From the present reciprocal inhibition data, it appears that the specific IgEs in the pool recognizing both peanut and almond proteins have a higher affinity towards peanut than almond epitopes, suggesting that peanut proteins are the sensitizing allergens, while almond proteins are the cross-reactive antigens. It is generally recognized that a cross-reactive antigen will have a lower affinity than the antigen that induced the antibody response [8]. In the case of hazelnut and pistachio, the reciprocal ELISA inhibition results suggest the existence of co-sensitizations to peanut, hazelnut and pistachio but moderate molecular cross-reactivities between peanut and these two tree nuts. These findings are supported by the low percent homology (generally below 50%) found between peanut and tree nut legumins, vicilins and albumins [11], which suggests the possibility for non-clinically relevant cross-reactions. Some previous studies were either contrasting or confirmatory to our results. Thus, de Leon et al. [12,16], in an Australian cohort, found, by using inhibition ELISA and a basophil activation assay, functional cross-reactivities between peanut and hazelnut and peanut and almond, while Noorbakhsh et al. [89], in an Iranian cohort, observed an insignificant IgE cross-reactivity between pistachio and peanut. The observed disparity in these findings indicate that cross-reactivity is patient-specific, as well as geographical area-specific. 

### 3.5. Identification of IgE Cross-Reactive Proteins by Reciprocal Inhibition Immunoblotting

**Figure 5:**
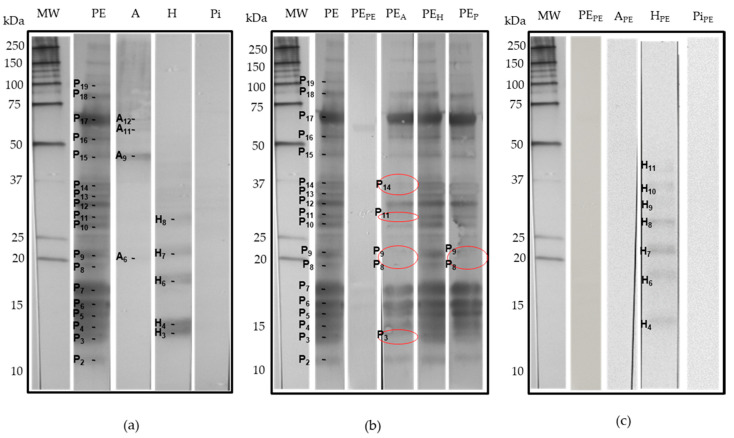
Reciprocal inhibition immunoblotting. (**a**) Peanut and tree nut extracts immobilized strips incubated with the uninhibited serum pool (PO): PE, peanut; A, almond; H, hazelnut and Pi, pistachio. (**b**) Peanut extract immobilized strips incubated with PO inhibited with each nut extract: PE, noninhibited with peanut; PE_PE_, inhibited with peanut; PE_A_, inhibited with almond; PE_H_, inhibited with hazelnut and PE_P_, inhibited with pistachio. (**c**) Tree nut extract immobilized strips incubated with PO inhibited with the peanut extract.

In order to identify more specifically cross-reactive IgE-binding proteins in the extracts of peanut and the studied tree nuts, reciprocal inhibition immunoblots were performed with the same serum pool used for the inhibition ELISA. At first, the incubation of tree nut extracts immobilized strips with the uninhibited serum pool (Figure 5a) revealed IgE binding to almond and hazelnut proteins but not pistachio proteins. For almond, four protein bands, named herein A6, A9 and A11, were compatible to storage proteins Pru du 2 (thaumatin-like protein, PR5), Pru du γ-conglutin and Pru du 10 (mandelonitrile lyase 2), respectively (Figure 1), while the IgE-binding A12 band did not correspond to any known allergen. For hazelnut, the uninhibited blot (Figure 5a) showed IgE binding to five protein bands possibly corresponding to allergens Cor a 2 (profilin, H3), Cor a 13 (oleosin, H4), Cor a 1 (pathogenesis-related protein PR-10, H6) and Cor a 9 (legumin 11S globulin, H7 and H8). Sensitization to Cor a 1 (PR-10 protein) in European patients has been associated to tree pollen allergies and generally results in mild allergic reactions, whereas sensitization to Cor a 9 is indicative of a systemic reaction to hazelnut [7]. 

As expected, serum IgE binding to peanut was almost completely abolished after the preincubation with its homologous inhibitor (peanut extract) (Figure 5b). Interestingly, preincubation of the serum pool with the almond extract attenuated the extent of binding to the immobilized peanut proteins, with the particular disappearance of several IgE-binding protein bands—namely, P3, P8, P9, P11, P13 and P14 (Figure 5b)—which were attributed to the subunits and protein fragments of the 11S globulin Ara h 3/4 and to 2S albumin Ara h 6 (Figure 1 and Table 2). On the reverse, the preincubation of the serum pool with the peanut extract strongly inhibited IgE binding to the immobilized almond proteins (Figure 5c), confirming its cross-reactive antigen status and not a primary sensitizing antigen. 

Preincubation of the serum pool with the hazelnut extract was unable to inhibit IgE binding to the immobilized peanut proteins (Figure 5b). However, when the serum pool was preincubated with the peanut extract (Figure 5c), binding to H4, H6, H7 and H8 remained uninhibited. Band H3, however, was strongly inhibited by the peanut extract. This band was described as profilin Cor a 2 (Table 2). This ubiquitous plant allergen presents a highly conserved structure, making it one of the highly cross-reactive panallergens from plant seeds [7], and could account for the mild observed cross-reactivity between peanut and hazelnut observed in this study. Interestingly, the peanut-inhibited blot revealed IgE binding to three more hazelnut protein bands: H9, H10 and H11 (Figure 5c), which were described as Cor a 9 (legumin 11S globulin) and Cor a 11 (vicilin 7S globulin) subunits. This might be explained by the fact that the capture of peanut and hazelnut cross-reactive sIgEs by the peanut protein resulted in an enrichment of the serum pool of the less-prevalent sIgE specific to these allergens (Cor a 9 and Cor a 11), thereby resulting in an increased immunoassay sensitivity of the test toward hazelnut. Altogether, these findings suggested the existence of both co-sensitization and cross-reactivity to hazelnut in this serum pool. A primary sensitization to hazelnut is highly possible for the two patients who exhibited high concentrations of (> 60 kU/L almond) of hazelnut sIgEs. A cross-reactivity between peanut and hazelnut was revealed by the inhibition ELISA but not by SDS-PAGE Western inhibition immunoblotting (Figure 5c), the latter being a denaturing technique that essentially targets primary linear structure epitopes, while the ELISA technique preserves, to a certain extent, the native tertiary structures of the proteins, which allows for the detection of conformational cross-reactive epitopes. This is consistent with the studies of sequence mapping, homology modeling and the conformational analysis of IgE-binding epitopes of peanut and hazelnut allergens, where the structural homology was observed between peanut and hazelnut allergens’ surface-exposed epitopes [13,14]. Moreover, previous research by Glaspole et al. [28] has demonstrated that hazelnut-specific T-cell lines obtained from patients with co-allergies to hazelnut and peanut proliferated upon stimulations with both hazelnut and peanut extracts and with Ara h 1 and/or Ara h 2 expressing both intracellular interleukin-5 (IL-5) and interferon-gamma (IFN-γ) responses. The authors suggested that a hazelnut and peanut co-allergy is associated with cross-reactive T-cell responses driven partly by cross-reactivity to the major peanut allergens Ara h 1 and Ara h 2. In another study, Akkerdaas et al. [90] reported a 56% shared sequence identity between recombinant peanut oleosin Ara h 10 and hazelnut oleosin Cor a 12 and a 69% shared sequence identity between Ara h 11 and Cor a 13, thereby possibly accounting for the observed molecular cross-reactions between hazelnut and peanut.

Finally, the inhibition of IgE binding to peanut with the pistachio extract resulted in the disappearance of the bindings to bands P8 and P3 attributed to peanut legumin Ara h 3/4, suggesting the presence of cross-reactivity between Ara h 3/4 and the pistachio homologous protein. In their study, Noorbakhsh et al. [89], using sera from three pistachio Iranian allergic subjects preincubated with a crude peanut extract, found no cross-reactivity between the pistachio and peanut allergens by the inhibition ELISA but showed a limited inhibition by the Western blot experiment. In our study, when the pistachio extract was immobilized on the blot, no IgE-binding was found with both the uninhibited or peanut-inhibited serum pool, indicating here again that the observed structural similarities of the conformational surface-exposed IgE epitopes between peanut and tree nut legumins are important in the observed IgE cross-reactivity between peanut and tree nut allergens [9,13,14]. The comparison of linear protein sequences alone cannot accurately predict the potential for cross-reactivity between peanut and nut allergens, as conformational protein epitopes may also play a role [11]. 

## 4. Conclusions

In this study, the pattern of sensitization to peanut allergens of a cohort of Canadian peanut-allergic children showed a typical North American sensitization profile, with intense reactivity to major peanut storage proteins corresponding to Ara h 2, Ara h 6, Ara h 1 and/or Ara h3. These allergens are generally indicative of severe/systemic reactivity. The results of the reciprocal inhibition ELISA experiments showed limited IgE cross-reactivities between peanut and the selected tree nuts, with, most likely, peanut being the sensitizing allergen and tree nuts the cross-reactive one. In the case of hazelnut and pistachio, a coexisting primary sensitization to these two nuts was demonstrated in the serum pool. Reciprocal inhibition immunoblotting further revealed that protein bands with molecular features compatible to known allergens from the storage protein families 2S albumin, 7S vicilin and 11S legumin could possibly be responsible for the observed IgE cross-reactions between peanut and the studied tree nuts in this cohort of allergic individuals. This study further highlighted that protein linear sequence homologies alone cannot accurately predict the cross-reactivity between peanut and tree nut allergens, as conformational epitopes play an important role in the observed IgE cross-reactivity. Although the findings of this study contribute to a better understanding of the molecular basis of peanut and tree nut cross-reactivities, further confirmation of protein/allergen identities by mass spectrometry is recommended. Additional studies allowing epitope structural and functional characterizations and differentiations between a clinically relevant and irrelevant cross-reaction are also needed to improve the diagnosis, management and treatment of peanut and tree nut allergies.

## Figures and Tables

**Table 1:** Clinical and sensitization profiles of the peanut-allergic children.

Patient No.	Age (years)	Sex	Symptoms to Peanut ^1^	SPT (mm)			Specific IgE (kU/L)			
				Negative Control	Histamine	Peanut Extract	Peanut	Almond	Hazelnut	Pistachio
1	3	M	eczema	0	4	8.0	˃100	0	0	0
2	4	M	eczema	0	4	8.5	˃100	1.13	˃100	4.36
3	3	M	eczema	0	3.5	5.0	˃100	1.44	3.32	13.4
4	12	M	skin rash and eye swelling	0	5	12.0	˃100	4.28	16.0	3.58
5	5	M	urticaria	0	3	14.5	˃100	8.73	60.2	3.95
6	12	M	Urticaria and eye swelling	0	5	15.0	˃100	0.67	0.48	0.21
7	13	M	ocular pruritus, eye swelling, urticaria and rhinorrhea and, 2 hours later, cough, dyspnea and wheezing	0	4	14.0	˃100	˂0.1	0.67	4.93
8	9	M	skin rash, swelling, cough, dyspnea and vomiting	0	3	8.5	˃100	0.534	0.878	12.6
9	9	M	urticaria	0	3	6.0	˃100	1.69	6.41	3.46

^1^ Allergic reaction symptoms are reported for peanut. Sensitivities to other tree nuts were found through specific immunoglobulin E (IgE) testing. Patients were avoiding tree nuts.

**Table 2:** Potential correspondence of resolved peanut, almond, hazelnut and pistachio proteins to known allergens.

Peak #	Allergen ^1^	Protein Family	Molecular Weight on SDS-PAGE ^2^ (kDa)	Reference
	**Peanut (*Arachis hypogea*)**			
P1	Ara h 9	Nonspecific lipid-transfer protein (LTP)	9.8	[55]
P2	Ara h 5	Profilin	12-15	[56]
P3	Ara h 6	Conglutin 2S albumin	14.2	[33]
P4	Ara h 7	Conglutin 2S albumin	15.8	[57]
P5	Ara h 10	Oleosin	16	[58]
P6	Ara h 2.01	Conglutin 2S albumin	17	[34]
P7	Ara h 2.02	Conglutin 2S albumin	19	[59]
P8/P9	Ara h 3/4	Legumin 11S globulin basic subunit	23–25	[60]
P10-P14	Ara h 3	Legumin 11S globulin acidic subunit	30–37	[32]
P15	Ara h 3	Legumin 11S globulin acidic subunit	42–45	[60,61]
P16	Ara h 3	Legumin 11S globulin	60	[62,63]
P17	Ara h 1	Vicilin 7S globulin	64	[38]
P18	Ara h 10	Oleosin oligomers	67–85	[58]
P19		Lipoxygenase	95–100	[39]
	**Almond (*Prunus dulcis*)**			
A1	Pru du 5	Ribosomal protein	10	[44]
A2	Pru du 2S albumin	Conglutin 2S albumin	12	[64]
A3	Pru du 4	Profilin	14	[65]
A4	Pru du 1	PR-10 protein	17	[32]
A5	Pru du 6	Legumin 11 S globulin (Amandin)	20–22	[66]
A6	Pru du 2	PR-5 protein (Thaumatin-like protein)	26	[67]
A7	Pru du 8	Antimicrobial seed storage protein	31	[46]
A8	Pru du 6	Legumin 11 S globulin (Amandin)	40	[68]
A9	Pru du γ-conglutin	7S vicilin storage protein	45	[67]
A11	Pru du 10	Mandelonitrile lyase 2	60	[69]
	**Hazelnut (*Corylus avellana*)**			
H1	Cor a 8	Nonspecific lipid-transfer protein (LTP)	9	[47]
H2	Cor a 14	Conglutin 2S albumin	11	[70]
H3	Cor a 2	Profilin	14	[71]
H4	Cor a 13	Oleosin	14–16	[72]
H5	Cor a 12	Oleosin	17	[72]
H6	Cor a 1	Pathogenesis-related protein	17	[73]
H7-H10	Cor a 9	Legumin 11S globulin	21–25; 31–35	[47,74]
H11	Cor a 11	Vicilin 7S globulin	48	[47]
H12	Cor a 11	Vicilin 7S globulin	55	[75]
H13	Cor a 10	Luminal binding protein	70	[76]
	**Pistachio (*Pistachia vera*)**			
P_i_1/P_i_2	Pis v 1	Conglutin 2S albumin	7 (17)	[48]
P_i_3	Pis v 4	Mn superoxide dismutase	26	[77]
P_i_4	Pis v 2	Legumin 11S globulin	32	[52]
P_i_5	Pis v 5	Legumin 11S globulin	36	
P_i_6/P_i_7	Pis v 2	Legumin 11S globulin	53	[52]
P_i_8	Pis v 3	Vicilin 7S globulin	55 (59)	[78]

^1^ Sources: Available at http://www.allergen.org and Allergome Platform (available at http://www.allergome.org). ^2^ Molecular weights are reported from the International Union of Immunological (IUIS) allergen nomenclature website when available or from referred publications.
